# Chelating capture and magnetic removal of non-magnetic heavy metal substances from soil

**DOI:** 10.1038/srep21027

**Published:** 2016-02-16

**Authors:** Liren Fan, Jiqing Song, Wenbo Bai, Shengping Wang, Ming Zeng, Xiaoming Li, Yang Zhou, Haifeng Li, Haiwei Lu

**Affiliations:** 1 Faculty of Materials Science and Chemistry, China University of Geosciences (Wuhan), Wuhan, Hubei 430074; 2 Institute of Environment and Sustainable Development in Agriculture, Chinese Academy of Agricultural Sciences, Beijing 100081; 3 Hunan Modern Environment Technology Co., Ltd, Changsha, Hunan

## Abstract

A soil remediation method based on magnetic beneficiation is reported. A new magnetic solid chelator powder, FS@IDA (core-shell Fe_3_O_4_@SiO_2_ nanoparticles coated with iminodiacetic acid chelators), was used as a reactive magnetic carrier to selectively capture non-magnetic heavy metals in soil by chelation and removal by magnetic separation. FS@IDA was prepared via inorganic-organic and organic synthesis reactions that generated chelating groups on the surface of magnetic, multi-core, core-shell Fe_3_O_4_@SiO_2_ (FS) nanoparticles. These reactions used a silane coupling agent and sodium chloroacetate. The results show that FS@IDA could chelate the heavy metal component of Cd, Zn, Pb, Cu and Ni carbonates, lead sulfate and lead chloride in water-insoluble salt systems. The resulting FS@IDA-Cd and FS@IDA-Pb chelates could be magnetically separated, resulting in removal rates of approximately 84.9% and 72.2% for Cd and Pb, respectively. FS@IDA could not remove the residual heavy metals and those bound to organic matter in the soil. FS@IDA did not significantly alter the chemical composition of the soil, and it allowed for fast chelating capture, simple magnetic separation and facilitated heavy metal elution. FS@IDA could also be easily prepared and reprocessed.

According to an official report recently released by the China Ministry of Environmental Protection^1^, 19.4% of the arable land in China is contaminated with heavy metals, such as cadmium, mercury, and arsenic, and organic pollutants, such as polycyclic aromatic hydrocarbons, resulting in environmental problems and food safety concerns. For example, in the “Poison Rice Incident” of May 2013, the Food and Drug Administration of Guangzhou, China, found that 44.4% of rice and related products consumed in Guangzhou were contaminated with cadmium^2^. The restoration of soil over large areas is urgently needed. Several strategies to remove heavy metals, such as chemical leaching^3^, electrokinetic remediation^4^, and phytoremediation^5^,^6^, have been proposed^7^,^8^,^9^,^10^ and are being used on a trial basis in China.

The use of magnetic nanoparticles (MNPs) as adsorbent materials to address environmental problems has received increasing attention due to their unique metal-ion adsorption properties and facile separation from aqueous solutions using a magnet^11^,^12^,^13^,^14^. Fe_3_O_4_ MNPs with adsorptive functional groups on their surfaces are well suited to selectively extract heavy metal ions from wastewater or industrial effluents^15^,^16^,^17^ by capturing the metal ions in solution. The loaded nanoparticles can then be retrieved with a magnet, and the metal ions can be subsequently stripped from the MNPs. This process is sustainable because the MNPs are reusable and no hazardous chemicals are employed.

Recently, unique core-shell MNPs, specifically Fe_3_O_4_@SiO_2_ (FS), that have surface chelating groups were prepared via organic-inorganic and surface functionalization reactions^18^. FS particles coated with N-[(3-trimethoxysilyl)propyl] ethylenediamine triacetic acid were prepared and used to extract and separate rare-earth ions^18^. These materials have also been functionalized with amino, imino and sulfonic groups and used to selectively remove Pb(II) and Cr(VI) from aqueous solutions^19^. Porphyrin-functionalized FS particles were employed to detect, adsorb and remove aqueous Hg^2+^ ions^17^. Comparing surface-functionalized FS particles to MNPs shows that they not only have improved resistance to acidic environments during magnetic retrieval, but they also selectively adsorb some heavy metal ions, which can be easily desorbed later. To date, however, virtually all literature reports of heavy metal-ion separation by MNPs and FS particles with chelating surface groups focus on aqueous solution systems. In contrast, the capture of other chemical forms of heavy metals in solid-liquid systems and multi-phase soil systems has not been widely investigated.

In this study, we report the preparation of a novel reactive magnetic carrier made by coating the inorganic surfaces of a magnetic powder (FS) with iminodiacetic acid chelators (FS@IDA) via organic synthesis reactions. The resulting FS@IDA material quickly captured the heavy metals of insoluble Cd, Zn, Pb, Cu and Ni carbonates, lead sulfate and lead chloride via chelation, which made the suspending liquids of the water-insoluble salt systems limpid. Furthermore, Cd and Pb in contaminated topsoil near a lead and zinc refinery in Zhuzhou, Hunan Province, China, formed magnetic solid chelate complexes with this novel material and were subsequently removed from the soil using a magnet.

## Results and Discussion

### Preparation and characterization of the FS@IDA powder

**Figure 1:**
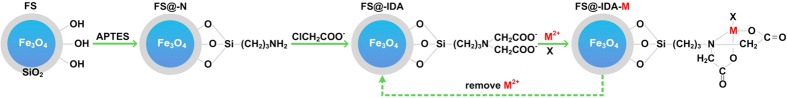
Schematic of the preparation and reprocessing of FS@IDA for the chelation and removal of heavy metal ions (M^2+^). FS, Fe_3_O_4_@SiO_2_; APTES, γ-aminopropyltriethoxysilane; FS@-N, surface-aminated FS; FS@IDA, magnetic solid trimethylene iminodiacetic acid chelator; FS@IDA-M, magnetic solid chelate complex; M^2+^, divalent heavy metal ion.

The preparation of FS@IDA is shown schematically in Fig. 1. The surface amination product FS@-N^20^,^21^ was prepared via the inorganic-organic reaction of FS particles^21^ with γ-aminopropyltriethoxysilane (APTES). Then, FS@IDA was prepared by the nucleophilic substitution reaction of FS@-N with sodium chloroacetate.

**Figure 2:**
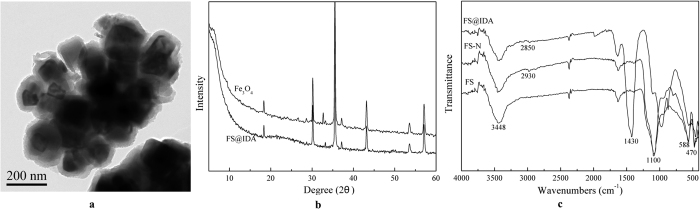
Structure and characterization of the FS@IDA material. (**a**) Transmission electron microscope image of FS@IDA. (**b**) X-ray diffraction patterns of Fe_3_O_4_ and FS@IDA. (**c**) Fourier transform infrared spectroscopy spectra.

Transmission electron microscope (TEM) images showed that the MNPs aggregates (dark gray) were coated with SiO_2_ membranes (light gray), indicating that the FS@IDA material had a multi-core, core-shell structure with a particle diameter of 0.3–3.0 μm (Fig. 2a). In the XRD pattern of FS@IDA, a broad peak was observed near 2θ = 23°, indicating the presence of non-crystalline SiO_2_ (Fig. 2b). The FTIR spectra of FS, FS@-N and FS@IDA (Fig. 2c) exhibited Si-O-Si vibration peaks at approximately 1100 and 470 cm^−1^ and a characteristic Fe_3_O_4_ adsorption peak at approximately 588 cm^−1^. The wide peak in the FS spectrum at about 3448 cm^−1^ was assigned to the vibration peak of hydrogen-bonded surface silanol groups. The intensity of this peak was weaker in the FS@IDA spectrum, suggesting that some of the silicon hydroxyl groups participated in the organic-inorganic reaction. The methylene vibrations of FS@-N and FS@IDA were observed at approximately 2930 and 2850 cm^−1^, respectively, and FS@IDA also exhibited a strong carboxyl adsorption peak at approximately 1430 cm^−1^. The results indicate that iminodiacetate groups were grafted on the FS surface. The amido group concentration of FS@-N was measured to be in the range of 0.035–0.067% (corresponding to 0.025–0.047 mmol·g^−1^) by the Kjeldahl nitrogen method and the pH of the solution was in the range 3 to 6. It was therefore assumed that the chelating group loading occurred in this amido group concentration range.

The sorption and acid desorption experiments using FS@IDA samples were repeated three times, and the nitrogen concentration of the initial sample and the acid desorption for the first, second and third times was measured continuously. They measured nitrogen concentration were 656, 646, 614 and 642 μg·g^−1^ (corresponding to 0.047, 0.046, 0.044 and 0.046 mmol), respectively. There was no decrease in the nitrogen concentration, which meant that the reduction of the effective group was relatively small, and the FS@IDA could be reused.

### Magnetic properties of FS@IDA

**Figure 3:**
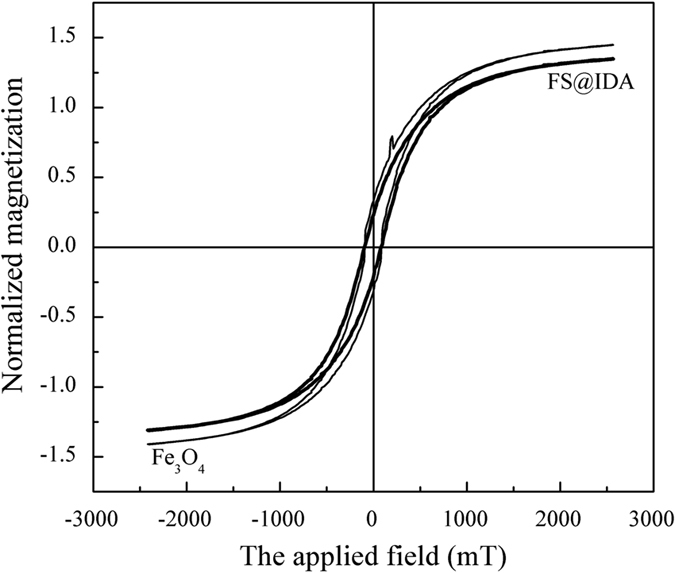
Magnetic hysteresis loop of FS@IDA.

The magnetic properties of FS@IDA (0.0455 g) were analyzed (Fig. 3). According to the hysteresis loop, the coercivity of FS@IDA was approximately 90 Oe, obviously indicating soft magnetic material. The saturation magnetization of FS@IDA was lower than that of Fe_3_O_4_, and declined by 7.6% in a small range due to the introduction of nonmetallic elements (C, N, O and Si), as well as the antimagnetic and covering effect of the nonmetallic elements. This effect also showed that the surface of Fe_3_O_4_ was coated completely. The slight decrease of saturation magnetization might be due to the effect of a thinner surface-coated modification layer.

The experiment measured the magnetic properties of fresh soil and soil treated with FS@IDA after magnetic separation. The recovery rate of FS@IDA was expressed as the ratio of dried FS@IDA (2.96 g) after magnetic separation to the amount added to the initial sample (3 g). The results show that 98.7% of the FS@IDA can be recycled. Errors were likely to have occurred during sample weighing and cleaning, so it is likely that this new material will be recyclable. Moreover, the main constituent of the material was iron, silicon and a novel reactive magnetic carrier created by coating the inorganic surfaces of a magnetic powder (FS) with iminodiacetic acid chelators (FS@IDA) via organic synthesis reactions, so there is no potential environmental risk.

### Effect of the pH on the amount of Cd^2+^ adsorbed on FS@IDA

**Figure 4:**
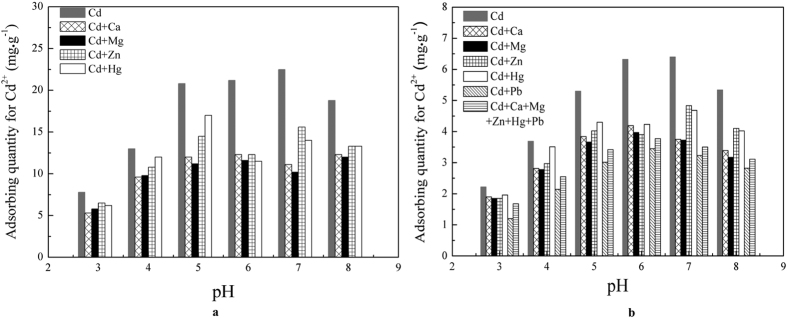
Effects of the presence of other cations on the quantity of Cd^2+^ adsorbed at different pH. (**a**) Initial Cd^2+^ concentration of 2.67 mM. (**b**) Initial Cd^2+^ concentration of 0.89 mM.

The number of solvated divalent metal ions adsorbed on FS@IDA was affected by the pH, adsorption time, FS@IDA quality, initial cadmium concentration and presence of other ions. Initially, 1 g of FS@IDA was added to 100 mL of 0.89 mM and 2.67 mM cadmium solutions, which were then maintained at room temperature for 12 h. The effects of the pH, the initial Cd^2+^ concentration and the presence of other ions on the amount of Cd^2+^ adsorbed were determined, and the results are shown in Fig. 4.

As shown in Fig. 4a,b, the amount of Cd^2+^ adsorbed increased with increasing pH and reached a maximum when pH was between 6 and 7. Above pH 7, the amount adsorbed decreased. The H^+^ concentration decreased with increasing pH, resulting in a decrease in the reactions between the carboxylate radicals, nitrogen atoms and H^+^ ions and increased chelation of Cd^2+^. However, the cadmium ion easily formed a hydrate under alkaline conditions, hindering its adsorption. When the initial Cd^2+^ concentrations were 0.89 mM and 2.67 mM, the maximum adsorption capacities of FS@IDA were about 6.40 mg·g^−1^ and 22.5 mg·g^−1^, respectively.

The adsorption behavior included both chemical and physical adsorption, and the maximal amount of Cd^2+^ adsorption was related to the initial Cd^2+^ concentration. Generally, the higher the initial concentration, the greater the adsorption quantity. So the adsorption of Cd^2+^ was higher than the nitrogen load. Where as, the nitrogen concentration of the sample was measured by a semi-micro Kjeldahl determination, and the results from this method were usually low, resulting in the adsorption capacity of the actual dynamic adsorption curve being larger than the nitrogen load, which denoted the quantity of the chelation group of the FA@IDA material.

Furthermore, Ca^2+^, Mg^2+^, Zn^2+^ and Hg^2+^ adsorption might compete with Cd^2+^ adsorption. The effects of Ca^2+^ and Mg^2+^ on the amount of Cd^2+^ adsorbed were the same and slightly greater than those of Zn^2+^ and Hg^2+^. In addition, when a solution containing equal amounts of Cd^2+^ and Pb^2+^ was used, the amount of Cd^2+^ adsorbed decreased by approximately 50%, showing that the presence of Pb^2+^ had a larger impact on cadmium adsorption than the other ions. These results indicate that FS@IDA can be used to selectively capture and separate solvated Cd^2+^ and Pb^2+^ ions.

### Chelating capture of several sparingly soluble heavy metals

Several insoluble carbonate powders (M = Cd, Zn, Pb, Cu, Ni), lead sulfate and lead chloride were added to 100 mL of aqueous solutions at pH 7. The heavy metal-ion concentrations in the solutions were 100 mg·L^−1^ (Cd: 0.89 mM, Zn: 1.53 mM, Pb: 0.49 mM, Cu: 1.57 mM, Ni: 1.69 mM). FS@IDA (3.5 g, the chelating group concentration was greater than 1.75 mmol·g^−1^) was added to the solution, which was intermittently stirred for 12 h at room temperature. The magnetic solid was separated using a magnet; the insoluble carbonate, sulfate and chloride powders were removed; and the suspension became clear (Supplementary Experiment S1).

In this study, the solubility of cadmium carbonate and the ion concentration were both low in the solution, so the effect of ionic strength on the solubility product could be ignored. In addition, the FS@IDA material was mainly used for soil heavy metal removal, and the conversion of heavy metal adsorption and precipitation would not result in a the pH change in the strong soil buffer system. Therefore, the aqueous solutions were adjusted to pH 7.

The solubility and concentration product constants correspond to the equilibrium conditions and to the actual experimental conditions, respectively. The results of the precipitate dissolution and heavy metal capture experiments are shown in Table 1. When an appropriate amount of FS@IDA was used, the Cd, Zn, Pb, Cu and Ni ions in the insoluble carbonate and the PbSO_4_ and PbCl_2_ compounds suspended in solution formed magnetic solid chelates with FS@IDA-M, allowing magnetic removal (Fig. 1), but the ions of the corresponding phosphate compounds did not form chelates with FS@IDA.

Based on solubility equilibrium theory, when the ion concentration product constant *K*_*sp*_′ is less than the solubility product constant *K*_*sp*_ of the insoluble salt, the insoluble salt dissolves.

The dissociation constants of H_2_CO_3_ in water are *K*_*a1*_ = 4.2 × 10^−7^ and *K*_*a2*_ = 5.6 × 10^−11^. The acid effect coefficient of CO_3_^2−^ is


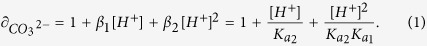



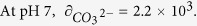


In this study, lead carbonate was used as an example to demonstrate precipitate dissolution.


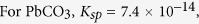



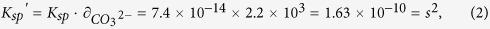


s = 1.28 × 10^−5^.


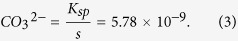


The [Pb^2+^]′ concentration of the solution was 1.01 × 10^−6^ M 12 h after the FS@IDA powder was added. Because


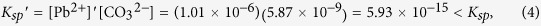


PbCO_3_ was dissolved in the solution, leading to the formation of FS@IDA-Pb.

The dissociation constants of H_3_PO_4_ in water are *K*_*a1*_ = 7.6 × 10^−3^, *K*_*a2*_ = 6.3 × 10^−8^ and *K*_*a3*_ = 4.4 × 10^−13^. The acid effect coefficient of PO_4_^3+^ is


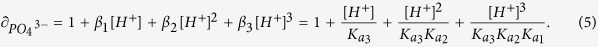



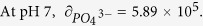


In this study, Zn_3_(PO_4_)_2_ was used as an example to demonstrate why precipitate dissolution did not occur.

With *K*_*sp*_ = 9.1 × 10^−33^, 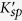 = 1.02 × 10^−25^, and 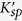 >* K*_*sp*_, the precipitate did not dissolve.

Lead sulfate and lead chloride were not hydrolyzed, and for these compounds, 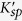 < *K*_*sp*_; therefore, these precipitates dissolved.

These results show that FS@IDA could promote the dissolution of some insoluble metal salts by capturing the metal ions with their chelating surface groups (Fig. 1), allowing for the targeted magnetic separation of multi-phase non-magnetic metal.

### Energy spectrum characterization of FS@IDA-Cd

**Figure 5:**
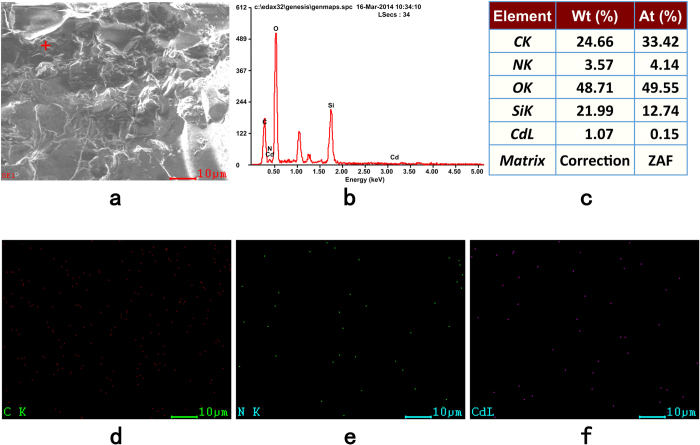
SEM-EDS of FS@IDA-Cd. (**a**) SEM image. (**b**) EDS point spectrum. (**c**) Relative elemental content at the corresponding point. EDS images showing the locations of (**d**) C, (**e**) N, and (**f**) Cd atoms.

In this study, the FS@IDA-Cd powder sample was obtained by magnetic separation. After drying the sample at 105 °C, its surface elements were analyzed with SEM-EDS^22^. The red dots (Fig. 5d) and green dots (Fig. 5e) in the full energy spectrum represent carbon and nitrogen atoms, respectively, which were introduced during the grafting reaction. The purple dots represent cadmium ions chelated by FS@IDA (Fig. 5f). Based on the energy spectrum, the nitrogen and cadmium loadings were found to be 3.57 and 1.07 wt%, respectively (Fig. 5c).

### Chelation and magnetic separation of Cd and Pb in soil

Heavy metals are found in different chemical forms in soils, and those forms have different activities in reactions such as dissolution, precipitation, coagulation and complex formation. Tessier *et al.*^22^ classified sediments and heavy metals in soils into different fractions; water-soluble, interchangeable, carbonate-bound, iron-manganese oxide-bound, organic-bound and residual fraction. The chemical stability of the different heavy metal fractions is as follows: residual >organic-bound >iron-manganese oxide-bound >carbonate-bound >interchangeable >water-soluble.

The topsoil near a lead and zinc refinery in Zhuzhou, Hunan Province, was chosen because it is representative of soils that are significantly contaminated by heavy metals. This factory specializes in heavy metals such as Pb, Zn, and Cu and their alloys and combines non-ferrous heavy metals such as Au, Ag, Cd, In, Ge, Ga, Se and Te, with chemical industry products such as vitriol. The cadmium and lead concentration was determined to be 10.91 ± 2.06 mg·kg^−1^ and 190.0 ± 33.2 mg·kg^−1^ (Table 2), respectively, using the GB/T17138–1997 standard method^23^. The heavy metal forms were analyzed using the Tessier method^22^.

To measure the amount of cadmium and lead removed from the soil, a slurry of 50 g of soil and 150 g of distilled water was prepared. The pH was adjusted between 6 and 7, and 5 g of FS@IDA was added to the slurry at room temperature. The magnetic solids were separated using a magnet after intermittent stirring for 7 d. The cadmium and lead concentration in the soil was reduced to 1.639 and 52.7 mg·kg^−1^ (Table 2), corresponding to removal percentages of 84.9 and 72.3%, respectively. These results suggest that the water-soluble, interchangeable, carbonate-bound and iron-manganese oxide-bound fractions of Cd and Pb (but not the organic-bound or residual fractions in the multi-phase soil system) could form the magnetic solid chelates FS@IDA-Cd and FS@IDA-Pb, which were subsequently separated using a magnet (Fig. 1 and Supplementary Experiment S2). This novel magnetic solid chelator as prepared in this study has potential for use in magnetic separation applications and in the removal of heavy metal contaminants from soil.

FS@IDA-Cd (1 g) was immersed in 1.0 M hydrochloric acid (100 mL) for 1 h, resulting in the desorption of more than 85% of the cadmium ions. The Cd^2+^ removal rate by FS@IDA was still greater than 55% after three adsorption-desorption cycles, indicating that this material can be recycled.

Related studies have reported that MNPs with high efficiencies can be used to remove heavy metals, such as Cd, Pb, Cu, Hg^24^,^25^,^26^, in aqueous solution. In this study, it was not emphasized that FS@IDA was a nanometer material. In fact, it was a multi-core, core-shell structure and the average particle size was not of nanomaterial particle size. Moreover, the nanoscale magnetic adsorption material is too small, while in the soil-liquid system and heterogeneous soil system, because of the fixation effect of soil clay, it is difficult for it to be completely separated, so the FS@IDA size was controlled in the range of a few microns to dozens of micrometers. The FS@IDA material was applied in a solid-liquid soil system and a heterogeneous soil system. The condition of the soil system was more complicated than in an aqueous solution. It was demonstrated that the FS@IDA had the potential to chelate sedimentary heavy metals (M) such as carbonates, sulfates, phosphates, and iron-manganese oxide-bound fractions, into soluble magnetic solids that were easy to separate magnetically.

### Chelation mechanism

The reaction between cadmium ions and FS@IDA illustrates the chelation mechanism. Both the nitrogen and oxygen atoms of the iminodiacetic acid chelating group have electron pairs that can coordinate with metal ions, resulting in a tridentate chelator structure. The FS@IDA surface has many alkyl iminodiacetic acid chelating groups that can coordinate with metal ions via the nitrogen and oxygen atoms to form stable four-coordinate, three-coordinate, and double five-membered ring chelates^27^. In addition, the cadmium ion has an 18-electron shell structure with an unoccupied orbital, a relatively small ionic radius and a relatively strong electronegativity and can therefore readily form six-coordinate or four-coordinate complexes^28^. Possible chelation reaction equations and the resulting FS@IDA-Cd structure showing the coordination of the FS@IDA chelating groups to the cadmium ion are shown in Fig. 1.

The magnetic solid chelate FS@IDA-Cd had cadmium ions coordinated with four atoms, the nitrogen and two oxygen atoms of a chelating group and solution species. H_2_O, OH^−^ or halogen ion in a double five-membered ring configuration. Therefore, it was assumed that the cadmium ions were captured via a chelating adsorption reaction on the FS@IDA surface or at the solid-liquid interface.

**Figure 6:**
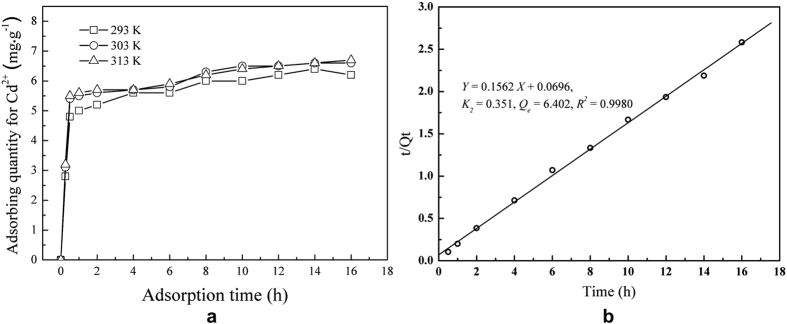
Amount adsorbed and adsorption dynamics at different temperatures. (**a**) Effects of the adsorption time on the amount of Cd^2+^ adsorbed on FS@IDA at different temperatures. (**b**) Data at 293 K were simulated using a quasi-secondary dynamics model, 
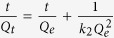
, where k_2_ is the adsorption rate constant, *Q*_e_ is the amount adsorbed at equilibrium (mg·g^−1^) and *Q*_t_ is the amount adsorbed at time t (mg·g^−1^).

The effect of the adsorption time on the amount of Cd^2+^ adsorbed on FS@IDA at different temperatures was measured. FS@IDA (1 g) was added to a 100 mg·L^−1^ Cd^2+^ solution (100 mL) and the pH was adjusted to 7. The temperature was set to 293, 303, or 313 K. The cadmium adsorption increased rapidly within the first 0.5 h, reaching over 75% of the maximum adsorption, indicating that these ions can be quickly captured by FS@IDA (Fig. 6a). The formation of chemical bonds during the adsorption process was the key factor affecting the secondary dynamic adsorption 
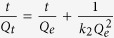
 and it was further assumed that cadmium was mainly adsorbed on FS@IDA via chelation (Fig. 6b).

## Conclusions

To achieve highly selective, efficient heavy metal removal from contaminated soil, a novel inorganic-organic hybrid material, magnetic solid chelator powder FS@IDA, was prepared. The inorganic FS particles displayed soft magnetism, and the organic alkyl iminodiacetic acid groups on the particle surface can capture heavy metals via chelation. Specifically, FS@IDA can coordinate with the heavy metals (M) of Cd, Zn, Pb, Cu and Ni carbonates, lead sulfate and lead chloride, which were insoluble in the aqueous systems tested, to form FS@IDA-M for magnetic separation. Only the organic-bound and the residual metal species in the soil cannot be chelated by FS@IDA. Moreover, FS@IDA-M could be easily reprocessed and recycled via acid treatment. This material has the potential to be used to selectively remove non-magnetic heavy metals from multi-phase soil systems under natural conditions via magnetic separation and could potentially be used to purify and manage large areas of farmland contaminated with heavy metals.

## Methods

### Materials

Fe_3_O_4_ powders (Tianjin Bodi Chemical Engineering Co., Ltd.,Tianjing, China), tetraethoxysilane (TEOS) (Tianjin Fuchen Chemical Reagent Factory, Tianjing, China), APTES (Hubei Wuda Organosilicon New Material Co., Ltd., Wuhan, China), and sodium chloroacetate (Tianjin Guangfu Fine Chemical Research Institute, Tianjing, China) were employed.

### Apparatus

TEM (Hitachi H-800), SEM (Hitachi SU-8010), XRD (Bruker AXS D8 Focus), Avatar 370 FTIR (US Nicolet), VSM (HH-10, Nanjing Nanda Instrument Plant), and Z-2000 AAS (Hitachi) instruments were employed.

### Preparation of FS

TEOS (9 g) and Fe_3_O_4_ powder (10 g) were stirred into 70 mL of anhydrous ethanol at 50 °C. Then, a solution of 12 mL of ammonium hydroxide, 30 mL of ethanol, and 14.4 mL of deionized water was added dropwise to the suspension under stirring, and the mixture allowed to react for 8 h. The product was separated using a magnet and washed with water. Finally, the resulting FS powder was vacuum-dried at 70 °C to a constant weight.

### Synthesis of FS-N

The FS powder (5 g) was added to 150 mL of anhydrous ethanol, and 1 g of APTES was added after stirring. The reaction solution was adjusted to pH 5. The reaction was maintained at 40 °C for 5 h. After the reaction, the product was separated using a magnet and washed with water. The magnetic FS-N particles were dried at 105 °C to generate the aminated multi-core core-shell structure.

### Carboxymethylation reaction on the FS-N surface

FS-N (5 g), 3 wt% sodium chloroacetate (100 mL), and a 10 v/v% ammonia solution (10 mL) were dissolved in 100 mL of distilled water. The reaction solution was maintained at 70 °C for 8 h. Then, the product was separated using a magnet and washed with distilled water. The FS@IDA powder was obtained by the nucleophilic substitution reaction of FS@-N with sodium chloroacetate after drying at 105 °C.

### The reutilization measurement of FS@IDA

Soil samples (30 g, 100-mesh seived) were added to 75 ml of distilled water and stirred for 10 min to prepare a soil suspension with a soil water ratio of 2.5. A columnar magnet with a magnetic field intensity of 5000 gauss was used to remove magnetic materials from the soil suspension. FS@IDA (3 g) was added to the soil suspension and stirred for 30 min. Then, the samples were magnetically separated after standing for 120 min, and the soil particles from the FS@IDA surface were washed with distilled water, dried at 70 °C and weighed

### Calculating the cadmium concentration in the solution

A cadmium solution with a given concentration was placed in a beaker and FS@IDA added. The pH was adjusted. After reacting at room temperature, the final product was separated using a magnet. The residual cadmium concentration in the solution was measured to calculate the amount adsorbed on FS@IDA and the cadmium removal rate.

The formula for calculating the amount adsorbed is


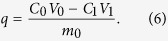


The formula for calculating the cadmium removal rate is


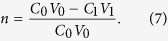


The initial concentration and volume of the cadmium solution are denoted by C_0_ (mg·L^−1^) and V_0_ (mL), respectively. The concentration and volume of the cadmium solution after the adsorption reaction are designated C_1_ (mg·L^−1^) and V_1_ (mL), respectively. The mass of FS@IDA used is m_0_ (g).

### Calculating the FS@IDA desorption rate

Saturated FS@IDA-Cd (1 g) was immersed in 100 mL of 1 M hydrochloric acid and the magnetic substances were separated using a magnet after the cadmium was allowed to desorb by stirring at room temperature for 1 h. The cadmium concentration in the solution was measured, and the desorption rate was calculated as:


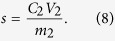


The concentration and volume of the cadmium solution after desorption were denoted by C_2_ (mg·L^−1^) and V_2_ (mL), respectively. The mass of cadmium adsorbed by FS@IDA was denoted m_2_ (g).

## Additional Information

**How to cite this article**: Fan, L. *et al.* Chelating capture and magnetic removal of non-magnetic heavy metal substances from soil. *Sci. Rep.* **6**, 21027; doi: 10.1038/srep21027 (2016).

## Supplementary Material

Supplementary Information

## Figures and Tables

**Table 1:** Results for the dissolution of several insoluble compounds suspended in solution and the corresponding metal ion capture.

Metal form	*K*_*sp*_ = [*M*^ 2+^]· 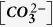	[M^2+^] = s (M)	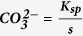	Ion concentration after adsorption [M^2+^]′ = s′ (M)	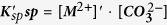	Comparison	Dissolution observed?
CdCO_3_	5.20 × 10^−12^	5.60 × 10^−4^	9.30 × 10^−9^	1.20 × 10^−4^	1.12 × 10^−12^	K_sp_′<K_sp_	yes
ZnCO_3_	1.40 × 10^−11^	1.75 × 10^−4^	8.00 × 10^−8^	1.75 × 10^−5^	1.40 × 10^−12^	K_sp_′<K_sp_	yes
PbCO_3_	7.40 × 10^−14^	1.28 × 10^−5^	5.78 × 10^−9^	1.01 × 10^−6^	5.93 × 10^−15^	K_sp_′<K_sp_	yes
CuCO_3_	1.40 × 10^−10^	5.56 × 10^−4^	2.52 × 10^−7^	3.17 × 10^−5^	7.99 × 10^−12^	K_sp_′<K_sp_	yes
NiCO_3_	6.60 × 10^−9^	3.82 × 10^−3^	1.73 × 10^−6^	6.10 × 10^−5^	1.56 × 10^−10^	K_sp_′<K_sp_	yes
**Metal form**	***K***_***sp***_** = [*****M***^**2+**^**]·[** 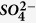 ]	[***M***^**2+**^**] = s (M)**	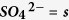	**Ion concentration after adsorption [M**^**2+**^**]′ = s′ (M)**	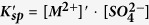	**Comparison**	**Dissolution observed?**
PbSO_4_	1.6 × 10^−8^	1.26 × 10^−4^	1.26 × 10^−4^	1.01 × 10^−6^	1.27 × 10^−10^	K_sp_′<K_sp_	yes
**Metal form**	***K***_***sp***_** = [*****M***^**2+**^**]·[** 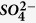 ]	**[M**^**2+**^**] = s (M)**	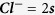	**Ion concentration after adsorption [M**^**2+**^**]′ = s′ (M)**	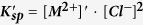	**Comparison**	**Dissolution observed?**
PbCl_2_	1.6 × 10^−5^	0.0252	0.0504	1.01 × 10^−6^	1.03 × 10^−8^	K_sp_′<K_sp_	yes

s is the solubility of the insoluble salt. *K*_*sp*_and 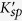 denote the solubility and concentration product constants, respectively.

**Table 2:** Total concentration and different fraction content of Cd, Pb, Cr, Zn, Hg and As in the soil sample (Mean ± SE, mg·kg^−1^).

Heavy metal concentration	Cd		Pb		As	Cr	Zn	Hg
	Before treatment	After treatment	Before treatment	After treatment				
Water-soluble fraction	0.012 ± 0.01	~0	0.058 ± 0.01	~0	0.292 ± 0.029	0.060 ± 0.08	0.736 ± 0.75	0.018 ± 0.006
Interchangeable fraction	2.758 ± 0.22		0.071 ± 0.013		0.363 ± 0.048	0.964 ± 0.097	2.846 ± 0.31	6.816 ± 1.38
Carbonate-bound fraction	3.381 ± 0.30		33.39 ± 3.01		0.309 ± 0.028	1.723 ± 0.20	73.83 ± 6.89	0.119 ± 0.02
Iron-manganese oxide-bound fraction	3.094 ± 0.34		67.92 ± 7.59		0.451 ± 0.056	4.512 ± 0.77	378.3 ± 43.1	0.202 ± 0.04
Organic-bound fraction	0.738 ± 0.067	1.639	9.435 ± 1.02	52.70	3.306 ± 0.45	18.68 ± 2.06	90.45 ± 9.9	10.23 ± 1.67
Residual fraction	0.917 ± 0.11		79.12 ± 8.96		47.33 ± 5.51	169.0 ± 18.80	164.9 ± 22.4	1.312 ± 0.19
Total content	10.91 ± 2.06		190.0 ± 33.2		50.75 ± 4.72	194.9 ± 49.9	660.9 ± 99.0	18.66 ± 2.75
