# Supplementary material for: Chelating capture and magnetic removal of non-magnetic heavy metal substances from soil
Source: Sci Rep. 2016 Feb 16;6:21027. doi: 10.1038/srep21027 (PMC4754707; doi:10.1038/srep21027)

**Supplementary Experiments**

**Title of the Manuscript**

Chelating capture and [magnetic](javascript:void(0);) removal of non-magnetic heavy metal substances from soil

**Author List**

Liren Fan, Jiqing Song, Wenbo Bai, Shengping Wang, Ming Zeng, Xiaoming Li, Yang Zhou, Haifeng Li & Haiwei Lu

**Supplementary Experiment Legend**

**Supplementary** **Experiment S1.** With the addition of FS@IDA, the suspension liquid containing CdCO_3_·H_2_O changed into FS@IDA-Cd and was intermittently stirred for 1 h at room temperature. The magnetic solids were separated using a magnet and the suspension became clear.

**Supplementary Experiment S2.**  FS@IDA (5 g) was added to the slurry (50 g of soil and 150 g of distilled water) at room temperature. The magnetic solids were separated using a magnet after intermittent stirring.

**Supplementary Experiments**

**Supplementary Experiment S1.**

With the addition of FS@IDA, the suspendsion liquid containing CdCO_3_·H_2_O changed into FS@IDA-Cd and was intermittently stirred for 1 h at room temperature. The magnetic solids were separated using a magnet and the suspension became clear.

**Step 1.**

A CdCO_3_·H_2_O solution of 100 mg·L^-1^ (Cd: 0.89 mM) was added to distilled water and intermittently stirred until fully mixed.


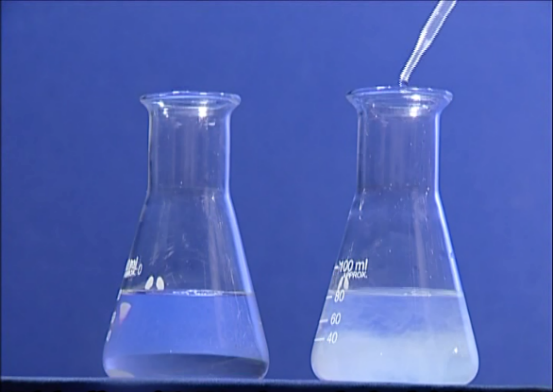

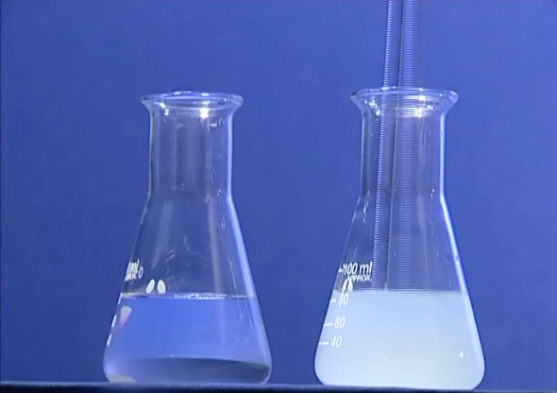


**Step 2.**

FS@IDA (3.5 g, with a chelating group concentration greater than 1.75 mmol·g^-1^) was added to the previous solution, which was intermittently stirred for 1 h at room temperature.


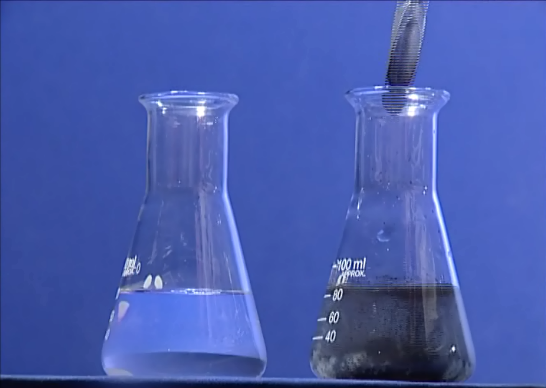

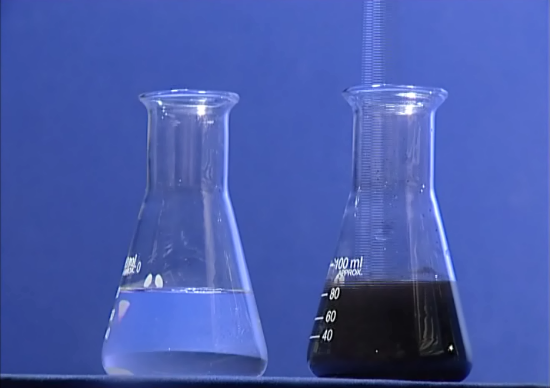


**Step 3.**

The magnetic solids were separated using a magnet and the suspension became clear.

**
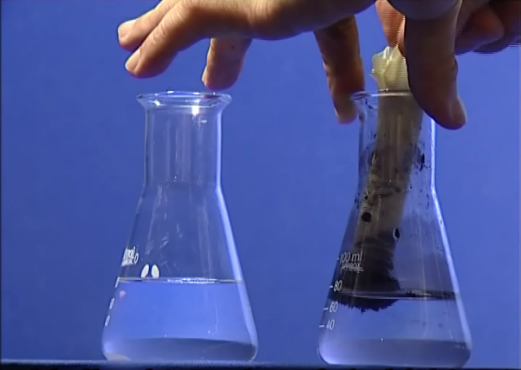
**

**Supplementary Experiment S2.**

FS@IDA (5 g) was added to the slurry (50 g of soil and 150 g of distilled water) at room temperature. The magnetic solids were separated using a magnet after intermittent stirring.

**Step 1.**

The 100-mesh soil samples (50 g) were added to 150 g of distilled water at room temperature, and stirred for 10 min to prepare a soil suspension with a water soil ratio of 3.


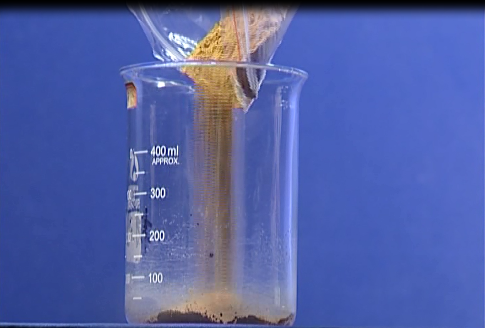

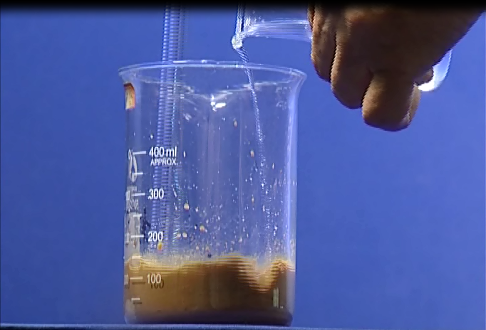

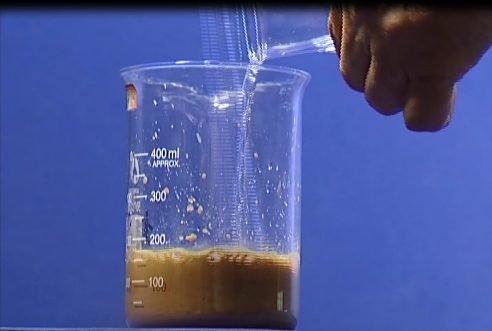


**Step 2.**

FS@IDA (5 g) was added to the previous slurry and intermittently stirred for 30 min.


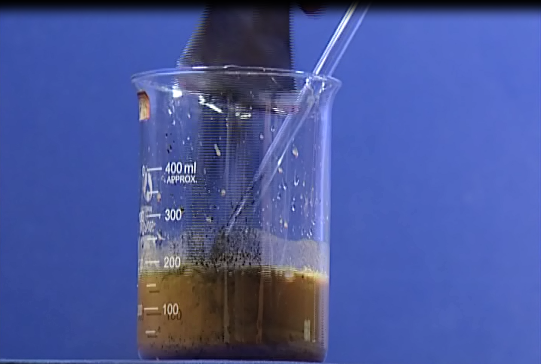

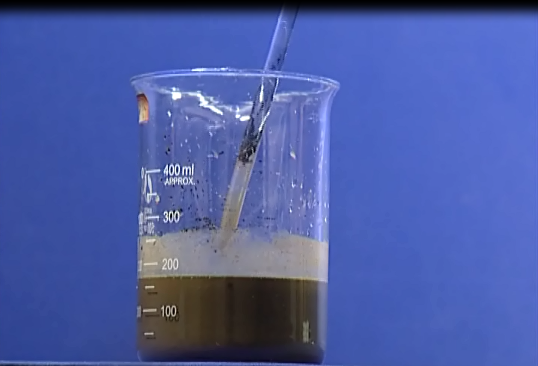


**Step 3.**

The magnetic solids were separated using a columnar magnet.


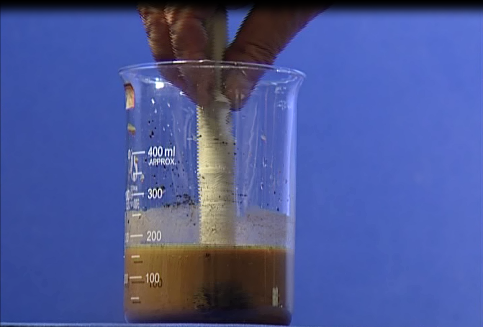

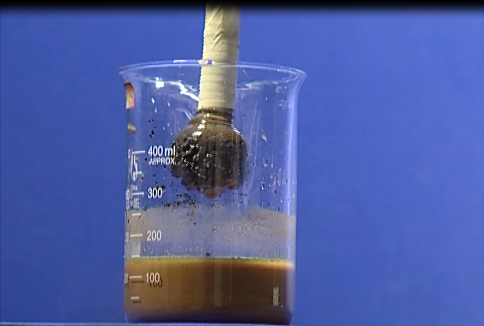

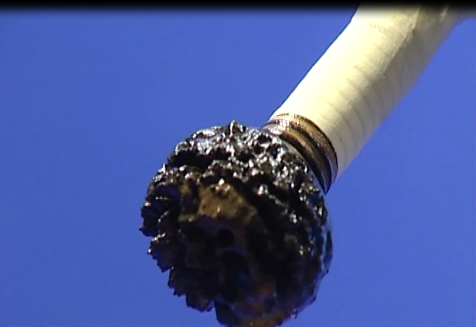

Supplement: Supplementary Information [file srep21027-s1.doc]
